# MiR-34a suppression targets Nampt to ameliorate bone marrow mesenchymal stem cell senescence by regulating NAD^+^-Sirt1 pathway

**DOI:** 10.1186/s13287-021-02339-0

**Published:** 2021-05-06

**Authors:** Chenchen Pi, Cao Ma, Huan Wang, Hui Sun, Xiao Yu, Xingyu Gao, Yue Yang, Yanan Sun, Haiying Zhang, Yingai Shi, Yan Li, Yulin Li, Xu He

**Affiliations:** 1 The Key Laboratory of Pathobiology, Ministry of Education, College of Basic Medical Sciences, Jilin University, 126 Xin Min Street, Changchun, Jilin Province People’s Republic of China; 2 The First Hospital, and Institute of Immunology, Jilin University, Changchun, 130021 China; 3 Department of Pathology, Zhongda Hospital, School of Medicine, Southeast University, Nanjing, 210009 China; 4 Department of Pathology, The First Affiliated Hospital, Henan University of Chinese Medicine, Henan, 450000 China; 5 Sichuan Academy of Medical Sciences and Sichuan Provincial People’s Hospital, Chengdu, 610072 China; 6 Division of Orthopedics and Biotechnology, Department for Clinical Intervention and Technology (CLINTEC), Karolinska Institute, Stockholm, Sweden

**Keywords:** Mesenchymal stem cell, Senescence, miRNA, miR-34a, Nampt, Regulation

## Abstract

**Background:**

Expansion-mediated replicative senescence and age-related natural senescence have adverse effects on mesenchymal stem cell (MSC) regenerative capability and functionality, thus severely impairing the extensive applications of MSC-based therapies. Emerging evidences suggest that microRNA-34a (miR-34a) has been implicated in the process of MSC senescence; however, the molecular mechanisms with regard to how miR-34a influencing MSC senescence remain largely undetermined.

**Methods:**

MiR-34a expression in MSCs was evaluated utilizing RT-qPCR. The functional effects of miR-34a exerting on MSC senescence were investigated via gene manipulation. Relevant gene and protein expression levels were analyzed by RT-qPCR and western blot. Luciferase reporter assays were applied to confirm that Nampt is a direct target of miR-34a. The underlying regulatory mechanism of miR-34a targeting Nampt in MSC senescence was further explored by measuring intracellular NAD^**+**^ content, NAD^+^/NADH ratio and Sirt1 activity.

**Results:**

In contrast to Nampt expression, miR-34a expression incremented in senescent MSCs. MiR-34a overexpression in young MSCs resulted in senescence-associated characteristics as displayed by senescence-like morphology, prolonged cell proliferation, declined osteogenic differentiation potency, heightened senescence-associated-β-galactosidase activity, and upregulated expression levels of the senescence-associated factors. Conversely, miR-34a suppression in replicative senescent and natural senescent MSCs contributed to diminished senescence-related phenotypic features. We identified Nampt as a direct target gene of miR-34a. In addition, miR-34a repletion resulted in prominent reductions in Nampt expression levels, NAD^+^ content, NAD^+^/NADH ratio, and Sirt1 activity, whereas anti-miR-34a treatment exerted the opposite effects. Furthermore, miR-34a-mediated MSC senescence was evidently rescued following the co-treatment with Nampt overexpression.

**Conclusion:**

This study identifies a significant role of miR-34a playing in MSC replicative senescence and natural senescence via targeting Nampt and further mediating by NAD^+^-Sirt1 pathway, carrying great implications for optimal strategies for MSC therapeutic applications.

## Background

Aging is a multifactorial process accompanied by conspicuous decline in quantity and quality of adult stem cells (SCs) and incremented susceptibility to age-related diseases [1]. Adult SCs, existing in almost all tissues and organs, are essential for maintaining tissue regeneration and homeostasis [2]. Nonetheless, SCs predispose to senesce with advancing age, which is one of the crucially precipitating factors during individual aging [1] and multiple age-associated disorders, such as atherosclerosis [3], diabetes mellitus [4], and neurodegenerative diseases [5].

Ascribed to wide variety of sources, multi-lineage differentiation potency, and plasticity in immunomodulatory capability, adult bone marrow-derived mesenchymal SCs (MSCs) have been one of the most invaluable candidates in tissue engineering and regenerative medicine [6, 7]. Before administration for transplantation, MSCs require extensive multi-passage in vitro expansion to acquire sufficient cells for treatment. However, even with highly self-renewal and regenerative capacity, MSCs will ineluctably undergo senescent state, which can be generally divided into two subsets. Cultivated primary cells in vitro undergo replicative senescence, which is telomere-initiated senescence [8, 9]. Another type of cellular senescence arising from in vivo chronological aging process of individuals, inescapably accompanied by distinctive senescence-related phenotypic characterization, is named as natural senescence [10]. These are considered as major impediments to applications in basic scientific research and clinical MSC-based therapeutic strategies. Hence, promising strategies to rejuvenate replicative and natural senescent MSCs merit urgent exploration.

MicroRNAs (miRNAs) have been participated in the modulation of multiple biological processes in organisms, encompassing cell proliferation and differentiation, cellular senescence, and energy metabolism [11, 12]. These RNA molecules influence gene expression by directly binding to the complementary sites presented in the 3′untranslated region (3′UTR) of target messenger RNAs (mRNAs), resulting in either posttranscriptional repression or degradation [11], given that MSC senescence is not only modulated by genetics, but also under the control of epigenetics [13]. As one of the vital mechanisms of epigenetic regulation, microRNA-34a (miR-34a), implicated in the senescence regulation network, has increasingly become a focus of intensive investigations [14, 15]. Nevertheless, the relationship between miR-34a and MSC senescence, and the detailed mechanisms are still far to be illuminated.

As proposed in the mammalian aging theory “NAD^+^ world,” nicotinamide adenine dinucleotide (NAD^+^), served as a key node, closely connects nicotinamide phosphoribosyltransferase (Nampt) and silent information regulator 2 ortholog (Sirt1) [16, 17]. Nampt, as the initiator, is the crucial rate-limiting enzyme involved in the NAD^+^ salvaging pathway, which can directly regulate the rate and yield of NAD^+^ biosynthesis, thus further affecting Sirt1 activity; Sirt1, as the effector, comes from a family of highly conserved NAD^+^-dependent protein deacetylases, and its activity is strictly controlled by NAD^+^ content [18, 19]. This tightly functional interplay between Nampt-mediated NAD^+^ biosynthesis and Sirt1 constitutes a systemic regulatory network, and we term it Nampt-NAD^+^-Sirt1 axis, which is of great significance to the maintenance of SCs function, cellular senescence, organismal aging, and homeostasis of energy metabolism [20, 21].

Nampt plays functional roles particularly in individual aging, cellular senescence, cell cycle maintenance, and cellular metabolism [22, 23]. Previously, we have validated that Nampt plays a pivotal role in the modulation of MSC natural and replicative senescence by mediating NAD^+^-Sirt1 signaling [10, 24]. However, it is still largely undefined the contribution of miR-34a to MSC senescence and whether the regulatory effects of Nampt on MSC senescence is modulated by miR-34a. Concomitantly, bioinformatics prediction implying that miR-34a has potential seed region binding sites with the 3′UTR of Nampt mRNA, we therefore hypothesized that miR-34a exerts functional effects on MSC senescence, and this process might be likely associated with Nampt-NAD^+^-Sirt1 axis. Here, we undertake a systematic study on the regulatory effects and molecular mechanism of miR-34a exerting on MSC replicative and natural senescence via gene manipulation and identified Nampt as its direct target gene.

## Materials and methods

### BM-derived MSC isolation and subculture

Primary MSCs of healthy, young (1–2 months old, Y) and old (15–18 months old, O) male Wistar rats were dissociated as previously described [10]. Briefly, single-cell suspensions of bone marrow cells were aseptically flushed out from the femurs and humeri with complete medium comprising Dulbecco’s Modified Eagle Medium with nutrient mixture F-12 (DMEM-F12, Gibco, Invitrogen, Carlsbad, USA), 10% heat-inactivated fetal bovine serum (FBS, Gibco, Invitrogen), and 1% penicillin/streptomycin solution (Gibco, Invitrogen). The medium was replenished every 2–3 days thereafter. Then, MSCs were detached by 0.25% trypsin-EDTA (Gibco, Invitrogen) and expanded at a ratio of 1:3 after reaching 80% confluence. MSCs at early passage 3, termed as P3MSCs and OMSCs, were obtained via serial cultivation in vitro from young and aged rats. And MSCs at late passages, P10MSCs used in the subsequent experiments were acquired from young P3MSCs via successive passages.

### Senescence-associated β-galactosidase activity assay

Senescent cell histochemical staining kit (Beyotime, Beijing, China) was used to evaluate SA-β-gal activity. Briefly, after, cells were fixed in fixation buffer for 15 min at RT, washed twice with phosphate-buffered saline (PBS), and incubated in Staining Solution Mix for 12 h while being sealed and protected from light at 37 °C without CO_2_. The reaction was stopped by PBS. Statistical analysis was performed by assessing the percentages of β-gal-positive cells in different microscopic fields.

### Gene expression analysis

Total RNA was extracted from tissues or MSCs using QIAzol Lysis Reagent from miRNeasy Mini kit (Qiagen, Hilden, Germany). After complementary DNA (cDNA) was synthesized with 1000 ng of miRNA using All-in-One™ miRNA First-Strand cDNA Synthesis Kit (GeneCopoeia, USA), the expression levels of miR-34 family members (miR-34a, miR-34b and miR-34c) were measured by real-time quantitative polymerase chain reaction (RT-qPCR) using miRNA-specific qPCR primers and All-in-One miRNA RT-qPCR Detection Kit (GeneCopoeia) in a 7300 Real-Time PCR System (Applied Biosystems, USA). U6 was amplified as a reference gene to normalize the relative expression of miRNA using the 2^−ΔΔCt^ cycle threshold method.

For detection of other gene expression, 500 ng of total RNA was used to synthesize cDNA with TransScript All-in-One First-Strand cDNA Synthesis SuperMix for qPCR (Transgen biotech, Beijing, China), and then relative amount of genes were measured by TransStart Top Green qPCR SuperMix (Transgen biotech) in ABI 7300 Real-Time PCR System. The rat specific primer sequences used are listed in Table 1. β-actin was amplified as a reference gene to normalize the relative expression of mRNA using the 2^−ΔΔCt^ cycle threshold method.

**Table 1:** Primers for RT-qPCR

Gene	Forward primers (5′-3′)	Reverse primers (3′-5′)
Nampt	AGGGGCATCTGCTCATTTGG	TGGTACTGTGCTCTGCCGCT
pl6^INK4^	AAACACTTTCGGTCGTACCC	GTCCTCGCAGTTCGAATC
p21^WAF1/CIP^	GACATCACCAGGATCGGACAT	GCAACGCTACTACGCAAGTAG
β-actin	GGAGATTACTGCCCTGGCTCCTA	GACTCATCGTACTCCTGCTTGCTG
snRNA U6	RmiRQP9003 GeneCopoeia, China	
rno-miR-34a-5p	RmiRQP0440 GeneCopoeia, China	
rno-miR-34b-5p	RmiRQP0980 GeneCopoeia, China	
rno-miR-34c-5p	RmiRQP0444 GeneCopoeia, China	

### Lentiviral transduction of MSCs

Prior to cell transduction, MSCs were seeded into 24-well plates at a density of 1.5 × 10^4^ per well. When the 40–50% confluence reached, cells were transduced with the purchased lentiviral particles encoding miR-34a and its LV-vector control miR-NC, anti-miR-34a, and the non-targeting anti-miR-NC (GeneChem, Shanghai, China) in the presence of 5 μg/mL polybrene (GeneChem) for 10-12 h. And as for miR-34a rescue assay, cells were co-transfected with lentiviral particles encoding rat Nampt or control vector (GeneChem) and miR-34a or miR-NC, respectively. Seventy-two to 96 h after the transduction, EGFP expression was monitored under a fluorescence microscope and then transduction efficiency was determined by RT-qPCR and western blot.

### Cell viability and proliferation assay

To evaluate cell proliferative ability, cell counting kit-8 (CCK-8) assay (Dojindo, Japan) was performed and PDT of MSCs was calculated as previously described [24]. Briefly, 1.0 × 10^3^ cells per well were seeded on a 96-well plate in triplicate. Then, 10 μl CCK-8 reagent was added to each well and mixed with the medium. After incubation at 37 °C for 2 h, the absorbance at 450 nm was measured by an ELISA plate reader (Thermo Labsystems, Finland) and monitored for 1 week. Cell growth curves were then produced to reflect cell growth kinetics. For PDT, 1.5 × 10^4^ cells were placed in a 24-well plate in triplicate and cultured. When cell confluence reached 80%, cells were harvested and recounted. PDT was calculated by the following equation: PDT = Ct/{In (Nf/Ni)/In(2)}; Ct = Tf − Ti, in which Ni is the number of initial seeded cells, Nf is the number of harvested cells, and Ct is the culture time.

### Cell cycle analysis

Cell cycle analysis was conducted using a Cell Cycle Detection Kit (KeyGEN BioTECH, Nanjing, China) as recommended by the manufacturer’s instructions. Briefly, 5 × 10^5^ cells of each group were fixed in 70% ethanol overnight at 4 °C. After rinsing 3 times with ice-cold PBS, cell pellets were treated with 100 μl RNaseA and incubated for 30 min at 37 °C. Next, 200 μl propidium iodide (PI) was added into the mixture for incubation at 4 °C for 20 min in the dark. DNA content was analyzed using the flow cytometry (BD Biosciences, USA), and the proliferative index (PI) and S-phase fraction (SPF) were compared and calculated with Cell Quest software.

### Osteogenic differentiation assay

Cells were cultivated in complete osteogenic culture medium when reached 70% confluence. A half osteogenic culture medium change was performed every 3 days. After the osteogenic induction of 2–3 weeks, cells were fixed with 4% paraformaldehyde for 30 min at 37 °C and then stained with alizarin red S working solution for 5 min to observe bone matrix mineralization. Further, to quantify mineralization, 10% cetylpyridinium chloride (Sigma-Aldrich, St. Louis, MO, USA) was added for 30 min at 37 °C. Respective absorbance values were measured by a kinetics ELISA reader (Thermo Labsystems, Finland) at 560 nm for final quantitative analysis.

### Target gene prediction of miRNA

Bioinformatics algorithms containing miRanda (Computational Biology Center at MSKCC, NY, USA), TargetScan (David Bartel Lab, Whitehead Institute for Biomedical Research, MA, USA), miRBase (University of Manchester, Manchester, UK), and PicTar (Rajewsky lab, NY, USA and Max Delbruck Centrum, Berlin, DE), and online database for miRNA target prediction (microRNA.org) were applied to predict the potential targets of miR-34a.

### Luciferase reporter assay

Luciferase constructs were constructed by inserting the full-length rat Nampt 3′ UTR, acquired from Imagenes in the psiCheck2 vector (Promega, Madison, USA). Cells were then seeded in 48-well plates and co-transfected with firefly luciferase reporter vector, miRNA expression vector in which 3′ UTR containing miR-34a seed sequence of Nampt, and the control vector containing Renilla luciferase (Promega) using Lipofectamine® 3000 (Invitrogen, Carlsbad, CA, USA). Luciferase enzymatic activity was measured 48 h after transfection using the Dual-Luciferase reporter assay system (Promega). And the firefly luciferase activity was normalized to Renilla luciferase activity for each sample.

### Western blot analysis

Total protein content was determined by a BCA Protein Assay Kit (Beyotime) after protein extraction using RIPA lysis buffer. Then, 25 μg of protein extracted from each sample were resolved by 10% SDS-PAGE gels and transferred to PVDF membranes (Millipore, Billerica, CA, USA) by electroblotting. The blotted membranes were blocked with 5% non-fat milk for 1-2 h at RT and then were probed with anti-Nampt (1:1000 dilution, BETHYL, USA) and anti-β-actin (1:1500 dilution, Abcam, UK) diluted in tris-buffered saline (TBS) overnight at 4 °C. After incubating with horseradish peroxidase-conjugated with anti-rabbit IgG secondary antibody (1:2000 dilution, Proteintech, USA), protein blots were visualized using an enhanced Electro-Chemi-Luminescence detection system (Amersham Biosciences, Piscataway, NJ, USA).

### Determination of NAD^+^ content and NAD^+^/NADH ratio

Intracellular NAD^+^ contents were quantified in accordance with manufacturer’s instructions, which were modified by scaling down assay volumes in all steps by half. Total 2 × 10^5^ cells were harvested and sorted directly into 200 μl of lysis buffer by NADH/NAD^+^ Extraction Buffer provided by NAD^+^/NADH Quantification assay Kit (BioVision, USA). The optical density was read at OD 450 nm using a multi-well spectrophotometer. Final NAD^+^ contents and NAD^+^/NADH ratio were calculated according to the standard curve created by NADH standards from the kit and the data obtained were normalized to the total cell number.

### Determination of Sirt1 deacetylase activity

Sirt1 deacetylase activity assay was performed by a SIRT1 assay kit (Sigma-Aldrich) following protocols approved by the manufacturer. Briefly, 20 μl protein extracted from different samples was gently blended with the mixture of 15 μl Assay Buffer and 5 μl NAD^+^ solution. Ten microliters of SIRT1 Substrate Solution was then added and incubated for 30 min at 37 °C. Thereafter, 5 μl developing solution was added into each well, and the whole cocktail of each group was incubated for another 10 min at 37 °C. Fluorescent intensity was measured at 460 nm (excitation 355 nm) using a plate reader, and Sirt1 activity was finally calculated according to a standard curve and normalized to protein content.

### Statistical analysis

All experiments were performed three times independently and data were presented as mean ± standard deviation. Statistical significance between groups was determined by using a two-tailed Student’s *t* test or a one-way ANOVA. *P* values indicated thusly: *P < 0.05, **P < 0.01, and ***P < 0.001 were considered statistically significant.

## Results

### Increased miR-34a expression levels along with expansion-mediated MSC replicative senescence and age-related MSC natural senescence

**Fig. 1:**
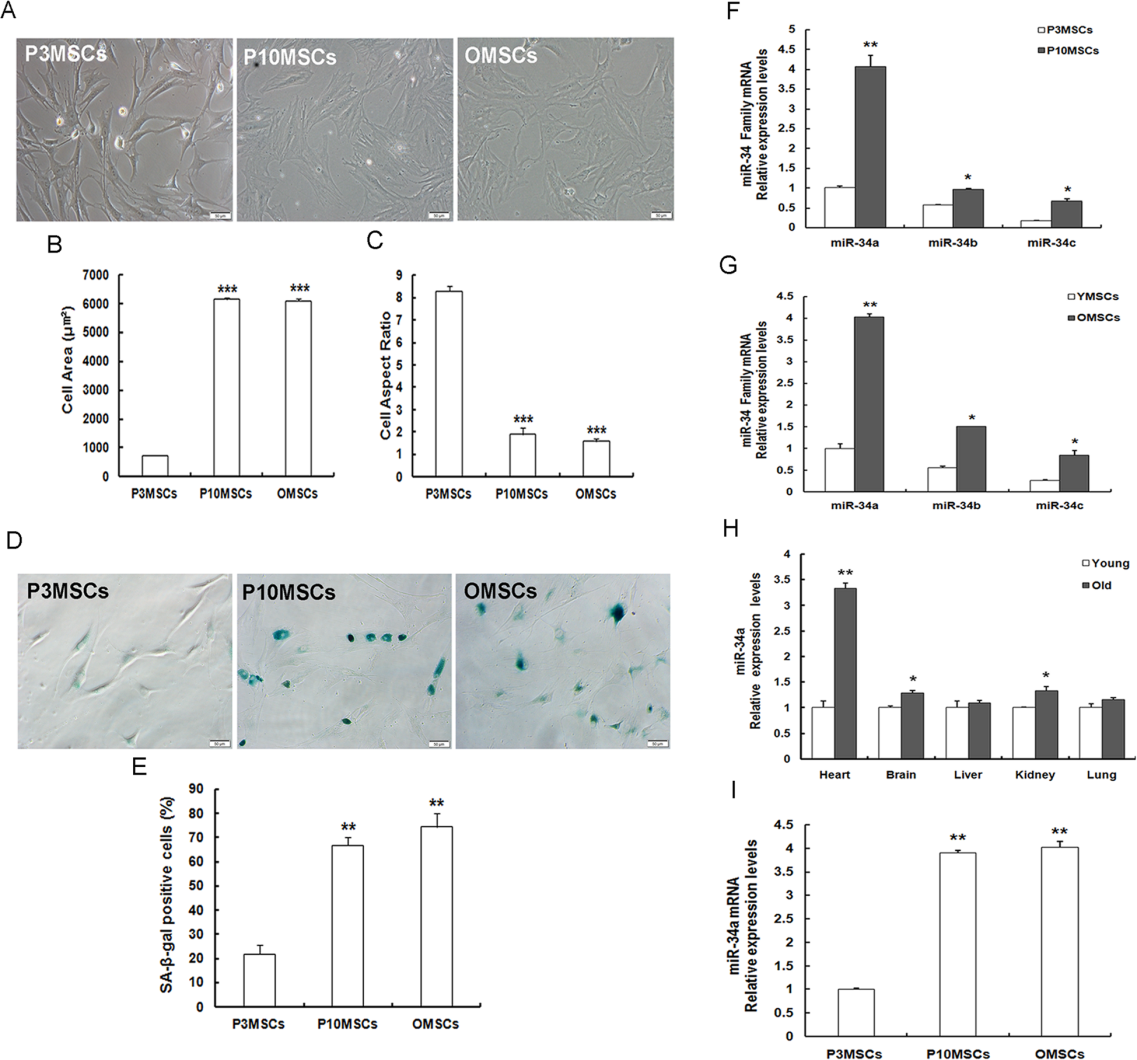
MiR-34a expression augments in senescent mesenchymal stem cells (MSCs). **a**–**c** Morphological evaluation of young and senescent MSCs. **a** Morphological features (scale bar = 50 μm) and quantitative analysis of cell surface area (**b**) and cell aspect ratio (**c**) of young P3MSCs (YMSCs), replicative senescent P10MSCs, and natural senescent OMSCs. **d**, **e** SA-β-gal staining (scale bar = 50 μm) (**d**) and quantitative analysis of the percentages of β-gal-positive cells (**e**) in P3MSCs, P10MSCs, and OMSCs. **f**, **g** Expression levels of miR-34 family members (comprising miR-34a, b, and c) in senescent P10MSCs (**f**) and OMSCs (**g**) compared to the young counterpart P3MSCs, respectively, assessed by RT-qPCR. **h** Examination of miR-34a expression levels by RT-qPCR in the hearts, brains, livers, kidneys, and lungs derived from young (1–2 months old, Y) and old (15–18 months old, O) Wistar male rats. **i** Detection of miR-34a expression levels by RT-qPCR. *n* = 3 independent experiments; *P < 0.05, **P < 0.01, ***P < 0.001 vs. P3MSCs, YMSCs

In the current study, young MSCs at early passage 3 (P3MSCs ,YMSCs) were obtained from young (1–2 months old, Y) rats, whereas senescent MSCs were either generated via serial cultivation in vitro to get MSCs at late passage 10 (P10MSCs) or derived from old (15–18 months old, O) rats to get natural senescent MSCs (OMSCs). Young P3MSCs, senescent P10MSCs, and OMSCs presented conspicuously different morphological features (Fig. 1a). P3MSCs displayed fibroblastic-like morphology with elongated and spindle-shaped cell bodies; meanwhile, both P10MSCs and OMSCs exhibited senescence-like morphology with flattened, enlarged, and irregular-shaped cell bodies, less stereoscopic and visible granules, and particles in the cytoplasm. Quantitative analysis for cell morphology revealed that cell surface area progressively increased (Fig. 1b), whereas the cell aspect ratio gradually decreased (Fig. 1c) in P10MSCs or OMSCs in comparison to P3MSCs. Cellular senescence-associated-β-galactosidase (SA-β-gal) staining has been considered as the gold standard to identify senescent cells in cell cultures [25, 26]. As displayed in Fig. 1d, only a small fraction of blue-stained cells was observed in P3MSCs, whereas this population was incremented in P10MSCs or OMSCs. And quantitative analysis (Fig. 1e) showed that the percentage of SA-β-gal-positive cells in P10MSCs or OMSCs was significantly more abundant than that in P3MSCs. These observations displayed that P10MSCs and OMSCs showed the senescent alterations unlike young P3MSCs/YMSCs, suggesting that replicative senescence appeared with extensive passages and natural senescence occurred with advancing age.

As miR-34 has been reported to be implicated in regulating senescence [27, 28], we next detected the expression of miR-34 family members (consisting of miR-34a, miR-34b, and miR-34c) by real-time quantitative polymerase chain reaction (RT-qPCR). And we observed the obviously elevated expression of the three miR-34 family members in MSC replicative senescence (Fig. 1f) and natural senescence (Fig. 1g), and among which miR-34a was the predominantly expressed one. Thence, we mainly dedicated to investigating the role of miR-34a in our subsequent experiments. MiR-34a expression levels in both tissues and cells derived from young and aged individuals were evaluated by RT-qPCR. Several tissues and organs including hearts, brains, livers, kidneys, and lungs obtained from young and aged rats were examined for miR-34a levels, and the results demonstrated that miR-34a expression levels were higher in all the tissues of old rats than those of the young ones, particularly in the hearts (Fig. 1h). Moreover, miR-34a expression levels incremented obviously in P10MSCs and OMSCs as compared to that in P3MSCs/YMSCs (Fig. 1i). The data indicated that miR-34a could be implicated in the modulation of expansion-mediated MSC replicative senescence and age-related MSC natural senescence.

### MiR-34a repletion accelerates young MSC senescence

**Fig. 2:**
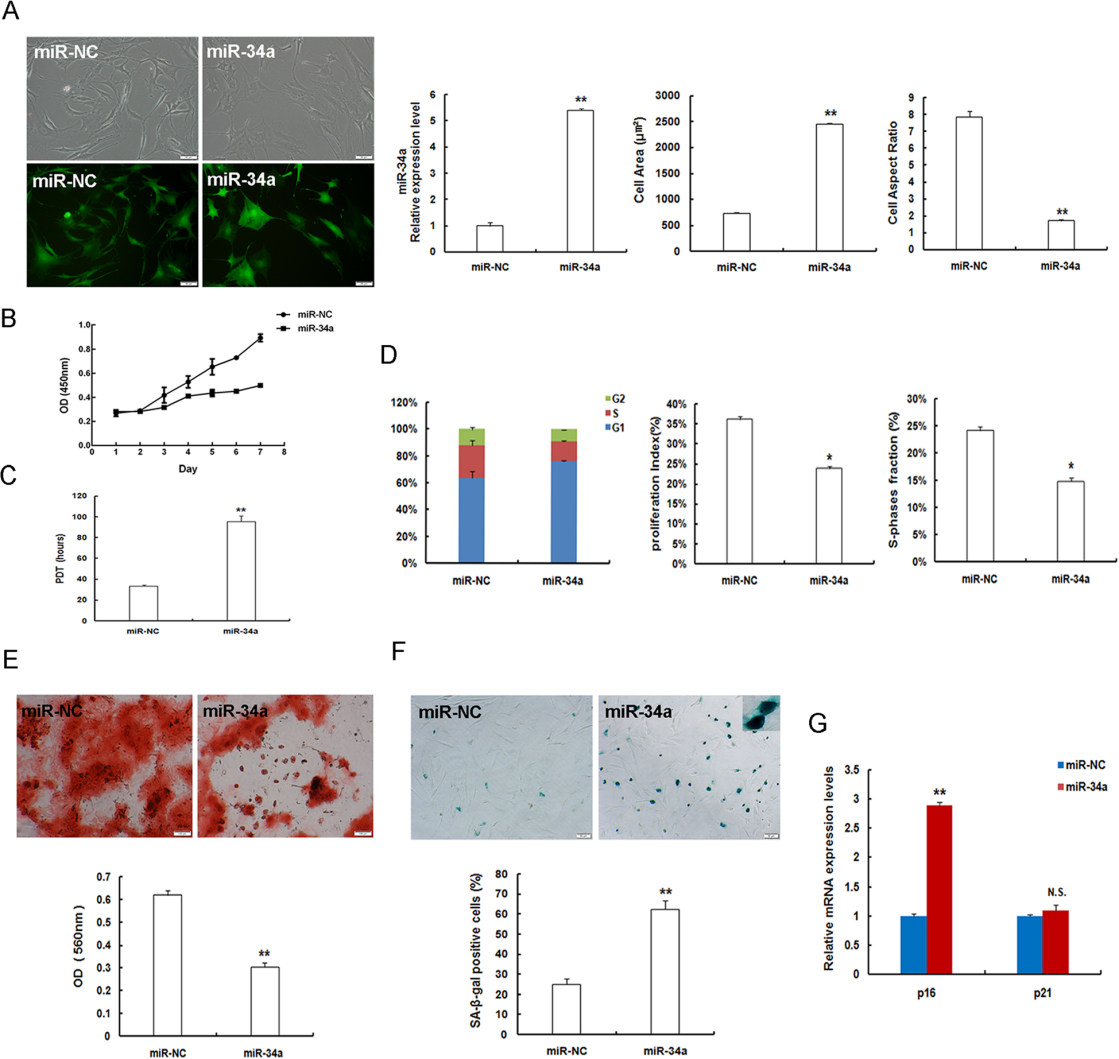
MiR-34a overexpression exacerbates senescence-associated variations of young P3MSCs. **a** Morphological features and EGFP expression under fluorescence microscope (scale bar = 50 μm). Demonstration of the transduction efficiency of miR-34a overexpression in P3MSCs by RT-qPCR. Quantitative analysis for cell surface area and cell aspect ratio in miR-NC and miR-34a over-expressed P3MSCs. **b** Cell growth curves in miR-34a over-expressed P3MSCs. **c** Cell population doubling time (PDT). **d** Detection of cell cycle by flow cytometry and analysis of proliferation index (PI) and S-phases fraction (SPF). **e** Determination of osteogenic differentiation in miR-34a over-expressed P3MSCs by Alizarin Red S staining (scale bar = 100 μm). **f** SA-β-gal staining (scale bar = 50 μm) and quantification of β-gal positive cell numbers. **g** Gene expression of the senescence-related factors p16^INK4a^ and p21^WAF1/CIP^ ; *n* = 3 independent experiments. *P < 0.05, **P < 0.01 vs. miR-NC, N.S., not significant

To corroborate the functional role of miR-34a play in MSC senescence, we first generated miR-34a over-expressed MSCs via transduction of lentiviral particles encoding miR-34a into young P3MSCs. Subsequently, the transduction efficacy was evaluated by observing enhanced green fluorescent protein (EGFP) expression and RT-qPCR analysis. As shown in Fig. 2a, mRNA levels of miR-34a expression were upregulated approximately 4.41-folds in miR-34a-replenished group in comparison with its negative control miR-NC group. Lentiviral-mediated miR-34a repletion made young P3MSCs switch into senescence-like morphology coupled with flattened and enlarged cell bodies, and less recognizable cell borders (Fig. 2a). The cell surface area was substantially increased, and the cell aspect ratio was noticeably decreased (Fig. 2a). In addition, cell proliferation as displayed by cell growth curves dampened (Fig. 2b) and the population doubling time (PDT) was conspicuously protracted (Fig. 2c), indicating a poor proliferation capacity after miR-34a overexpression. The percentages of each phase of cell cycle was analyzed and revealed that there was an obviously numerical increase of cells arrested in G1 phase, and the proliferative index (PI) and S-phase fraction (SPF) were much lower in the miR-34a over-expressed group than those in miR-NC group (Fig. 2d). Stem cells are not only characterized by self-renewal and cell proliferation capability, but are also fundamentally featured by multilineage differentiation. We next found that miR-34a over-expressed MSCs presented impaired osteogenic differentiation with diminished bone matrix mineralization, as assessed by Alizarin red S staining (Fig. 2e). Quantitative analysis demonstrated that osteogenic differentiation potential of P3MSCs was significantly attenuated following the upregulation of miR-34a expression. Furthermore, the percentage of SA-β-gal-positive cells accumulated extensively (Fig. 2f) in the miR-34a sufficient group. And the gene expression of senescence-associated factor p16^INK4a^ heightened markedly, whereas p21^WAF1/CIP^ mRNA levels showed no significant changes in young P3MSCs after miR-34a over-expressing treatment (Fig. 2g). Accordingly, these observations support the notion that miR-34a replenishment negatively affects and induces or promotes senescence-related variations of young MSCs.

### MiR-34a depletion alleviates expansion-mediated MSC replicative senescence

**Fig. 3:**
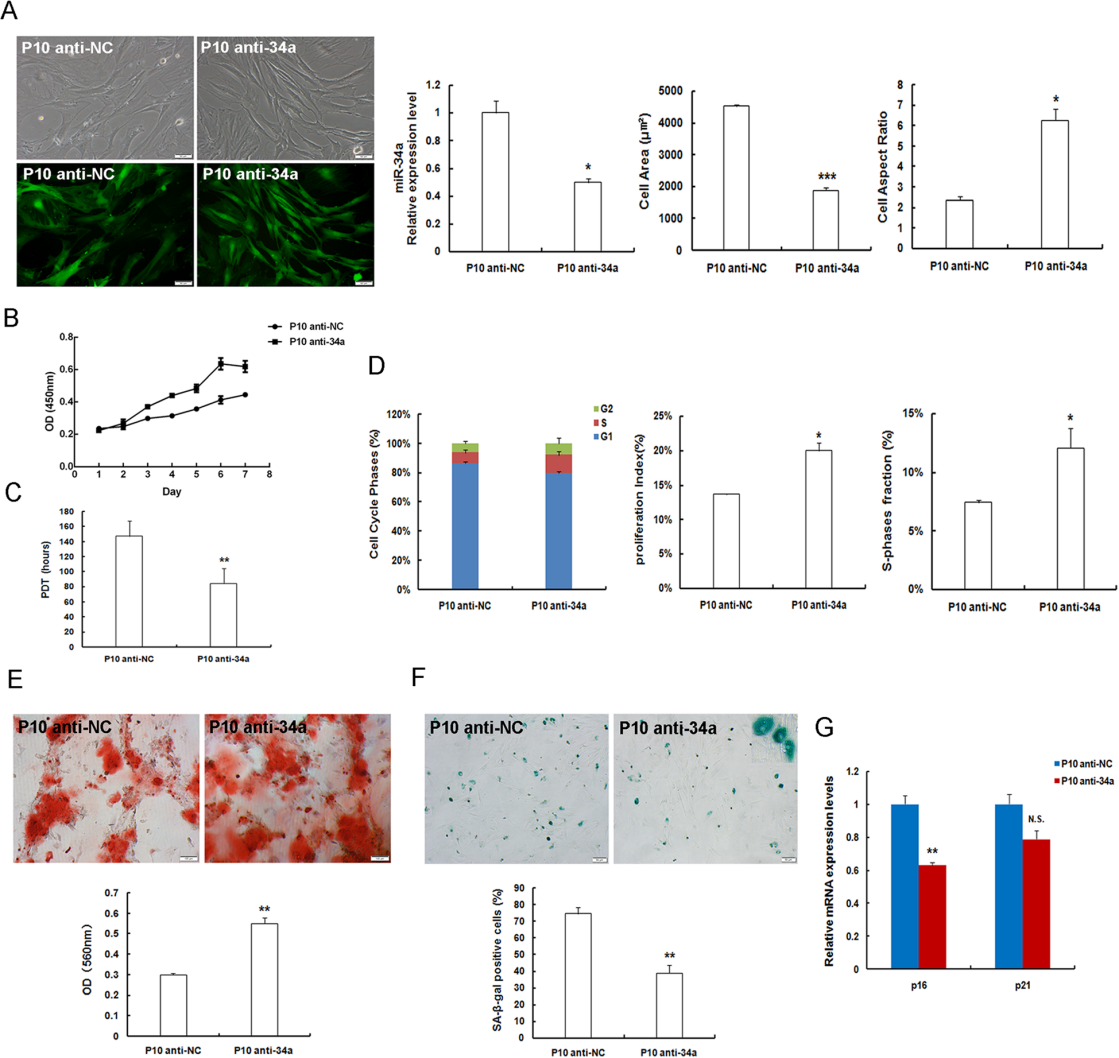
MiR-34a suppression alleviates MSC replicative senescence in P10MSCs. **a** Morphological features and the presence of EGFP-expressing P10MSCs (scale bar = 50 μm). Evaluation of the transduction efficacy of miR-34a depletion in P10MSCs by RT-qPCR. Quantification for cell surface area and cell aspect ratio in anti-NC and anti-34a expressed P10MSCs. **b** Logarithmic proliferation in miR-34a-deficient P10MSCs. **c** Population doubling time (PDT). **d** Assessment of cell cycle. **e** Observation of osteogenesis (scale bar = 100 μm) and quantification. **f** Cellular SA-β-gal staining (scale bar = 50 μm) and quantification. **g** Detection of mRNA expression levels of senescence markers p16^INK4a^ and p21^WAF1/CIP^ in replicative senescent P10MSCs after miR-34a suppression by RT-qPCR; *n* = 3 independent experiments. *P < 0.05, **P < 0.01, ***P < 0.001 vs. P10 anti-NC, N.S., not significant

With respect to the high levels of miR-34a expression exhibited in replicative senescent P10MSCs, we investigated whether abating miR-34a expression could attenuate MSC replicative senescence. For this purpose, P10MSCs were transduced with lentivirus expressing anti-sense miR-34a (anti-34a) and the lentiviral vector (anti-NC). And the transduction efficacy and loss of miR-34a expression in miR-34a-deficient P10MSCs were verified by observing EGFP expression and RT-qPCR. Notably, miR-34a expression at mRNA level was markedly reduced by nearly 50% in P10 anti-34a group as compared to that in P10 anti-NC group (Fig. 3a). And miR-34a deficiency led to the morphological alterations in P10MSCs from senescence-like enlarged and flattened cells to elongated and spindle-shaped ones with decreased cell area and increased cell aspect ratio (Fig. 3a). The cell growth became faster and cell proliferation capability was enhanced (Fig. 3b), whereas the PDT was shortened evidently (Fig. 3c). What is more, cell cycle analysis showed that there was a numerical decrease of cells arrested in G1 phase; both PI and SPF were much higher in the miR-34a-inhibited group than those in P10 anti-NC group (Fig. 3d). Of note, mineralized nodule formation in P10MSCs was obviously increased after abating miR-34a expression. Quantitative analysis indicated that osteogenic differentiation potency of P10MSCs was significantly improved by anti-miR-34a treatment in these replicative senescent cells (Fig. 3e). Furthermore, SA-β-gal staining and quantitative analysis demonstrated that SA-β-gal activity in miR-34a-deficient P10MSCs noticeably declined as compared to that in P10 anti-NC group (Fig. 3f). And the levels of p16^INK4a^ expression were dramatically downregulated, whereas the levels of p21^WAF1/CIP^ expression displayed no significant alterations in replicative senescent P10MSCs after miR-34a silencing (Fig. 3g). Taken together, these data manifested that miR-34a depletion can positively influence cellular senescence and contribute to diminished senescent phenotypes in replicative senescent P10MSCs, suggesting that miR-34a inhibition can alleviate or suppress expansion-mediated MSC replicative senescence.

### MiR-34a suppression mitigates age-related MSC natural senescence

**Fig. 4:**
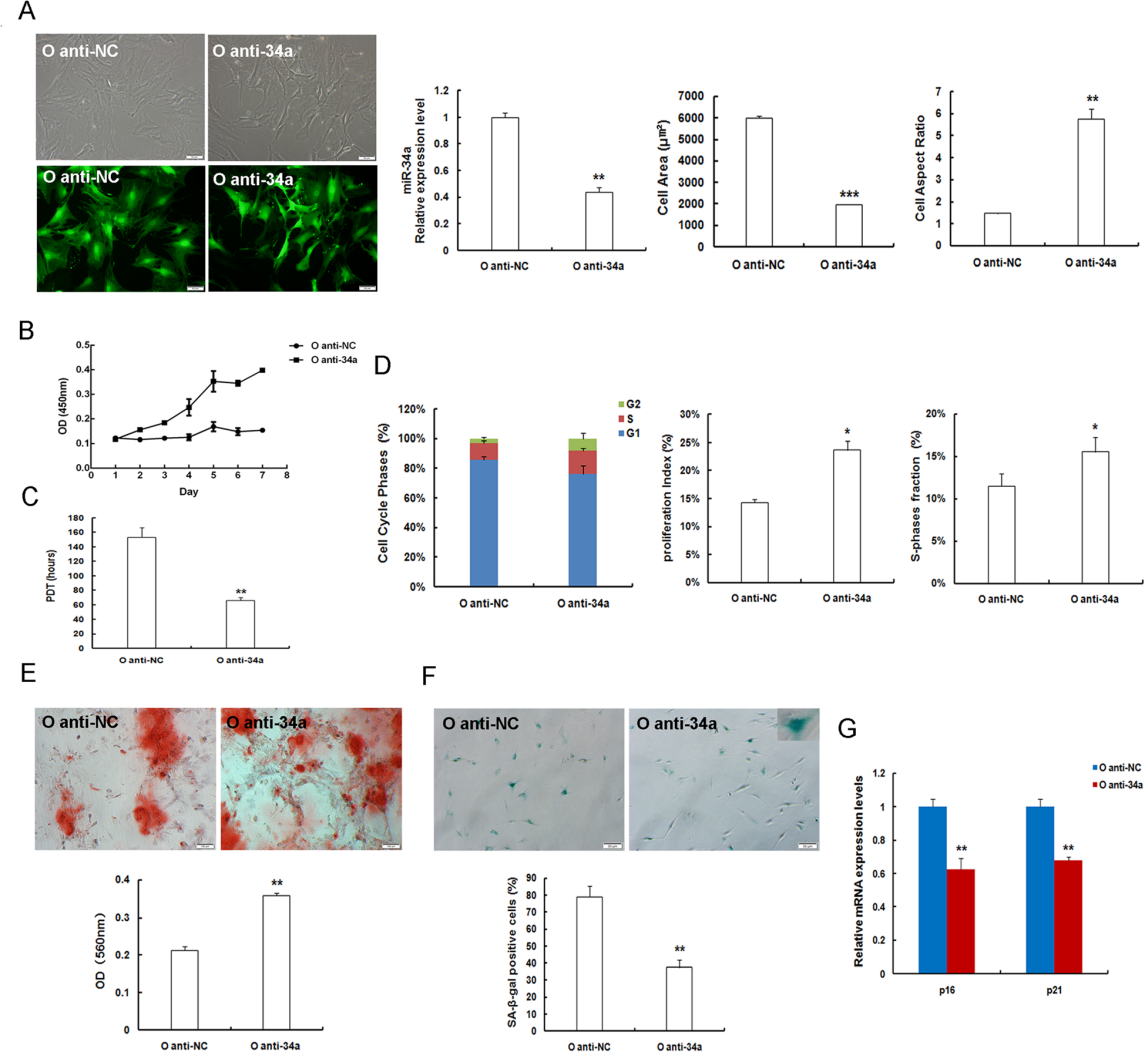
MiR-34a inhibition mitigates MSC natural senescence in OMSCs. **a** Representative images showing morphological appearance (scale bar = 50 μm) and EGFP expression. RT-qPCR expression analysis for transduction efficacy of miR-34a reduction in OMSCs. Quantification for cell surface area and cell aspect ratio in anti-NC and anti-34a expressed OMSCs. **b** Logarithmic proliferation in miR-34a-insufficient OMSCs. **c** Population doubling time (PDT). **d** Detection and analysis of cell cycle. **e** Osteogenic differentiation of MSCs (scale bar = 100 μm) and quantification. **f** SA-β-gal staining (scale bar = 50 μm) and quantification. **g** Determination of mRNA expression levels of the senescence markers p16^INK4a^ and p21^WAF1/CIP^ in natural senescent OMSCs after miR-34a inhibition by RT-qPCR; *n* = 3 independent experiments. *P < 0.05, **P < 0.01, ***P < 0.001 vs. O anti-NC

Given that miR-34a also highly expressed in OMSCs, we wondered whether miR-34a is also participated in regulating MSC natural senescence. Thence, OMSCs were generated through transducing with lentivirus expressing anti-34a and the lentiviral vector anti-NC (Fig. 4a). As indicated by the results of RT-qPCR analysis, miR-34a expression of OMSCs was successfully depleted at mRNA levels (Fig. 4a). Moreover, cellular morphology was noticeably altered by miR-34a deficiency. These flattened and enlarged senescent cell bodies in O anti-NC group became substantially slenderer and more elongated, and the cell borders became more distinct. The cell surface area prominently decreased, whereas the cell aspect ratio markedly increased (Fig. 4a). On the other hand, miR-34a-suppressed OMSCs grew faster (Fig. 4b), and the PDT was shortened (Fig. 4c). The percentage of cells arrested in G1 phase was also obviously decreased by miR-34a depletion, and both PI and SPF were obviously boosted in O anti-34a group when compared to the O anti-NC group (Fig. 4d). As shown in Fig. 4e, matrix mineralization in OMSCs was largely augmented following miR-34a inhibition. Quantitative analysis exhibited that osteogenesis in natural senescent OMSCs were remarkably augmented after suppressing miR-34a expression. In addition, SA-β-gal staining and quantitative analysis manifested that the ratios of SA-β-gal-positive cells in miR-34a-repressed OMSCs were reduced after the anti-miR-34a treatment (Fig. 4f), and other senescence-associated biomarkers, both p16^INK4a^ and p21^WAF1/CIP^ mRNA expression displayed obviously downward trend in miR-34a-repressed OMSCs (Fig. 4g). Together, these studies suggested that miR-34a inhibition is implicated in the positive regulation of MSC natural senescence resulting in the alleviation of senescence-associated changes in natural senescent OMSCs, indicating that miR-34a suppression can ameliorate or repress age-related MSC senescence.

### Inverse correlation between miR-34a and Nampt-mediated NAD^+^ Synthesis and Sirt1 deacetylase activity

**Fig. 5:**
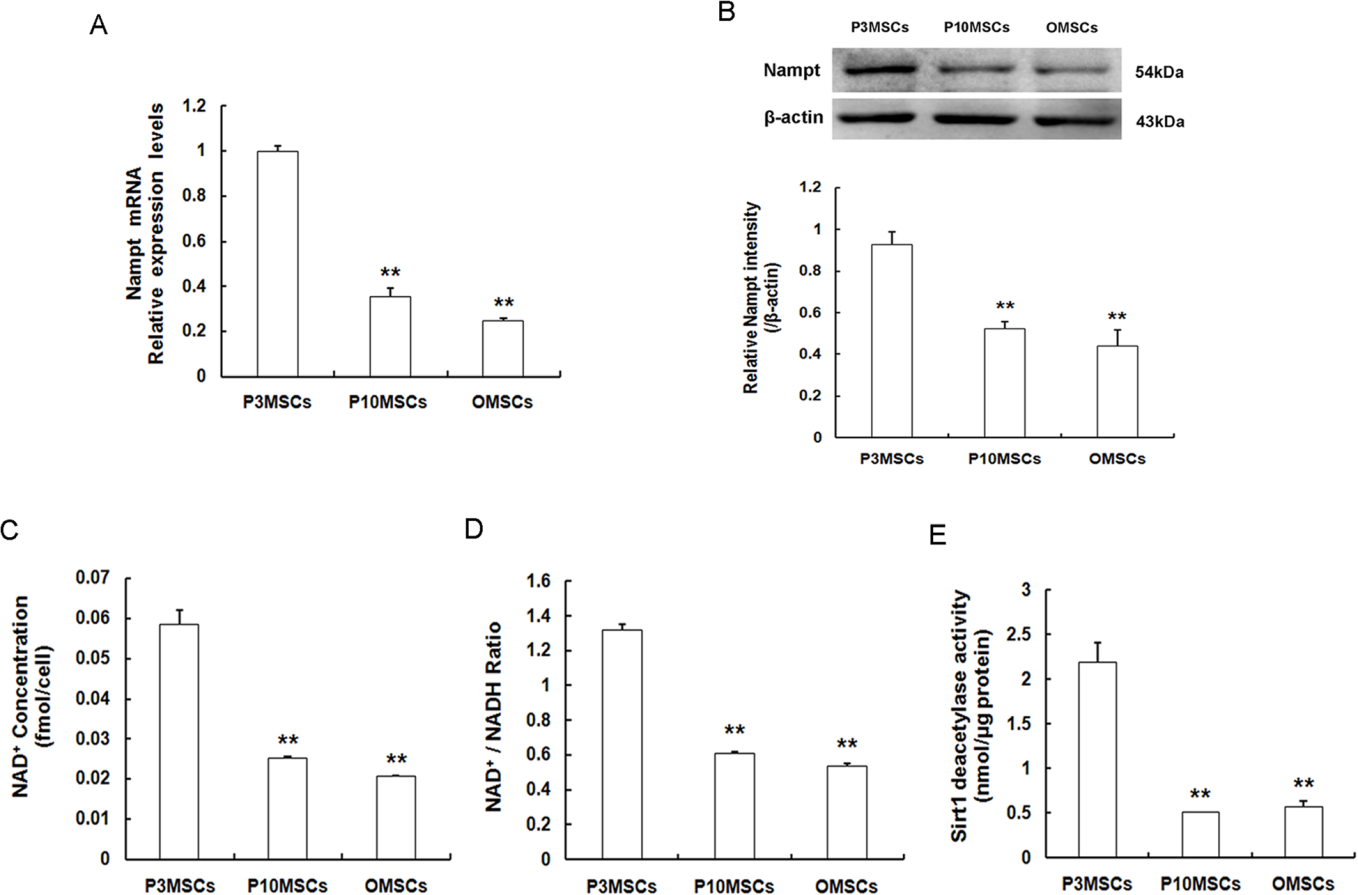
Measurement of Nampt expression and NAD^+^-Sirt1 signaling in replicative and natural senescence. **a** Determination of Nampt mRNA expression levels by RT-qPCR. **b** Determination of Nampt protein expression levels by western blot. **c**–**e** Determination of basal NAD^+^ level (**c**), NAD^+^/NADH ratio (**d**), and Sirt1 deacetylase activity in P3MSCs, P10MSCs, and OMSCs, respectively; *n* = 3 independent experiments. **P < 0.01 vs. P3MSCs

In our preliminary studies, it has been authenticated that senescence is highly associated with passage-dependent and age-dependent reduction of Nampt expression, suggesting that Nampt plays a crucial role in the regulation of expansion-mediated MSC replicative senescence and age-related MSC natural senescence [10, 24]. Herein, we examined the levels of Nampt expression in P3MSCs, P10MSCs, and OMSCs by RT-qPCR (Fig. 5a) and western blot (Fig. 5b). And as expected, we confirmed that Nampt expression at both mRNA and protein levels were obviously dampened in senescent P10MSCs and OMSCs in comparison with the young counterpart P3MSCs. Of note, intriguingly, we found that in contrast to the reduction of Nampt expression along with the replicative (Fig. 1i) and natural senescence (Fig. 1j), miR-34a expression contrarily incremented in these senescent MSCs, implying that there is likely to be a converse correlation existed between miR-34a and Nampt expression. Moreover, guided by the mammalian aging theory “NAD^+^ world” and our previous studies, we subsequently detected NAD^+^-Sirt1 signaling pathway. The data displayed that intracellular NAD^+^ content (Fig. 5c), NAD^+^/NADH ratio (Fig. 5d), and Sirt1 activity (Fig. 5e) in senescent P10MSCs and OMSCs were remarkably lower than those in young P3MSCs. Consequently, these results manifested that there might exist a converse relationship between miR-34a and Nampt-mediated NAD^+^ synthesis and Sirt1 activity, by which we hypothesized that miR-34a may directly target Nampt and then modulate the NAD^+^-Sirt1 signaling pathway, thus exerting effects on MSC senescence.

### Nampt identified as a direct target gene of miR-34a

**Fig. 6:**
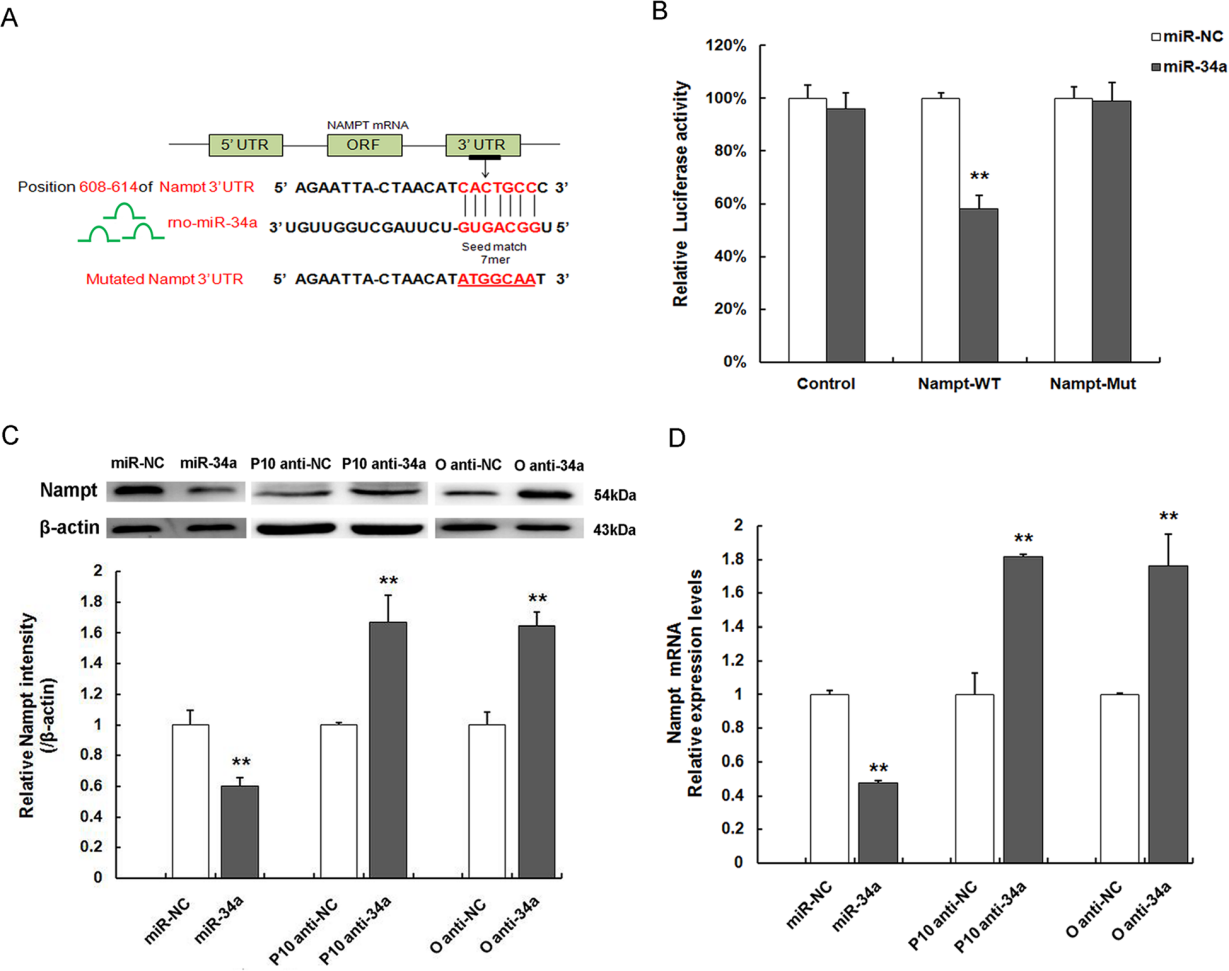
MiR-34a directly targets Nampt. **a** Schematic representation of the predicted complimentary base pairing between miR-34a and the 3′UTR of Nampt and the mutated binding site of putative miR-34a seed sequence. **b** Analysis of luciferase activity in cells co-transfected with miR-NC or miR-34a and plasmid containing the 3′ UTR of the wild type (WT) Nampt or a mutated (Mut) Nampt sequence in luciferase reporter assay. **c** Detection of Nampt protein expression levels by western blotting. **d** Detection of Nampt mRNA expression levels by RT-qPCR after interfering with miR-34a expression; *n* = 3 independent experiments. **P < 0.01 vs. miR-NC, P10 anti-NC, or O anti-NC

To assess our hypothesis, we identify whether Nampt is the putative target mRNAs of miR-34a applying several bioinformatics algorithms. The bioinformatics analysis revealed that there existed potential seed-matching sites between miR-34a and the 3′UTR of Nampt mRNA, indicating that Nampt was a candidate target gene for miR-34a (Fig. 6a). For the purpose of determining whether Nampt is a potential target gene of miR-34a, we performed luciferase reporter assay. To this end, luciferase reporter constructs were generated, in which the Nampt 3′UTR or the putative miR-34a seed sequence binding site mutated from 5′-CACTGCCC-3′ to 5′-ATGGCAAT-3′ (Fig. 6a) is inserted behind the luciferase gene. As displayed in Fig. 6b, miR-34a repletion significantly suppressed the luciferase activity, manifesting that miR-34a can bind to the Nampt 3′UTR, whereas no effects were observed as binding sites of miR-34a was mutated. What is more, the intensity of Nampt protein expression, as quantified from the immunoblots, displayed that in comparison with their respective control, miR-34a repletion could obviously downregulate Nampt expression in P3MSCs at protein levels, while miR-34a suppression in P10MSCs and OMSCs could evidently upregulate Nampt protein expression (Fig. 6c).

Congruent with the observations at protein levels, the mRNA levels of Nampt expression following miR-34a or anti-miR-34a treatment by RT-qPCR analysis showed a markedly decrease in miR-34a-over-expressed P3MSCs, while the increase was much more pronounced in miR-34a-repressed P10MSCs and OMSCs (Fig. 6d). Together, we thus infer that miR-34a directly targets Nampt and negatively regulates Nampt.

### MiR-34a-induced senescence rescued evidently by Nampt replenishment and mediated by NAD^+^-Sirt1 signaling pathway

**Fig. 7:**
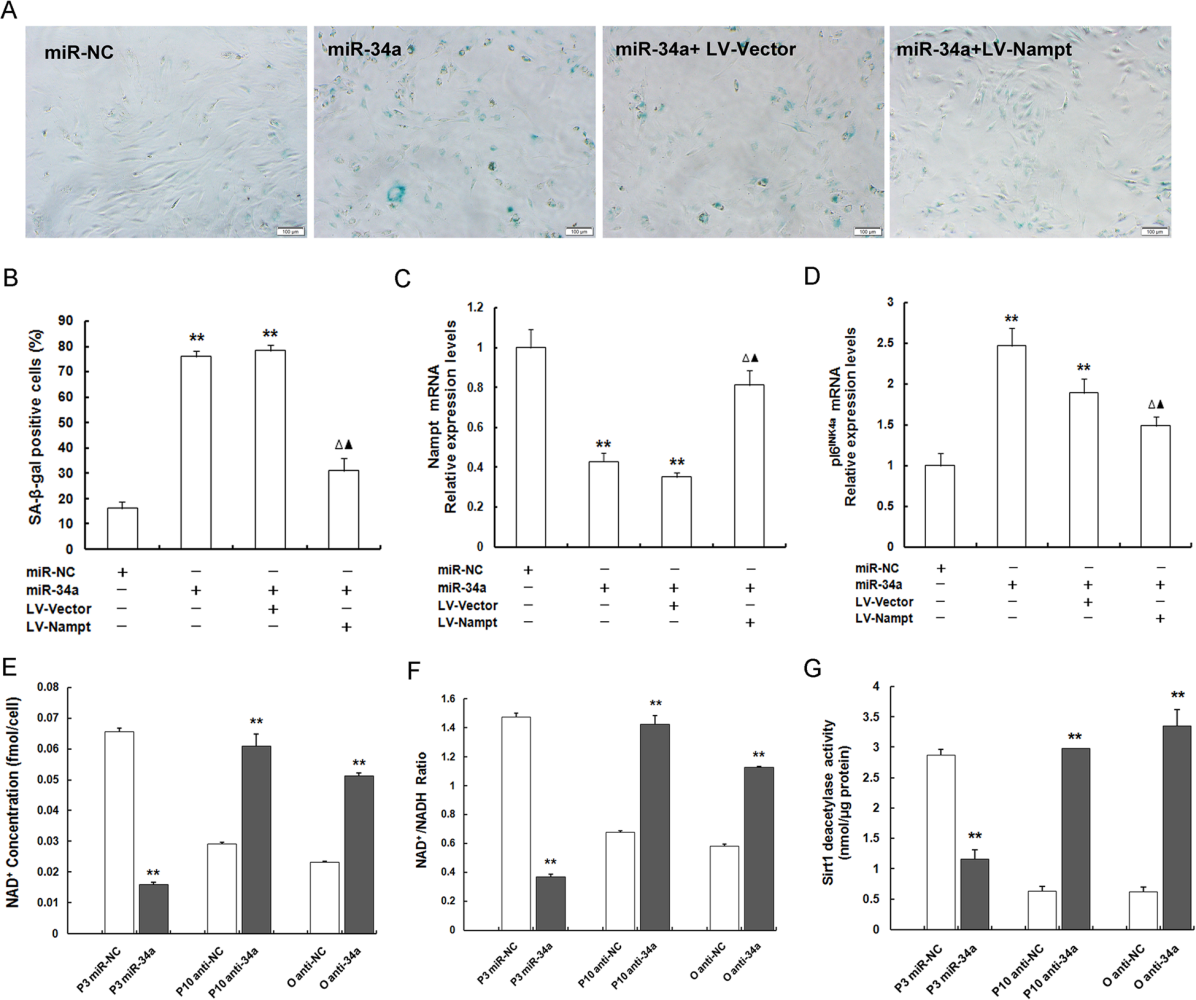
MiR-34a level alterations exert on MSC senescence via Nampt-NAD^+^-Sirt1 axis. **a**, **b** SA-β-gal staining (scale bar = 100 μm) (**a**) and quantification (**b**) in P3MSCs treated with miR-NC, miR-34a, miR-34a plus LV-Vector or miR-34a plus LV-Nampt. **c**, **d** Detection of mRNA expression levels of Nampt (**c**) and senescence-related factors p16^INK4a^ (**d**) by RT-qPCR after interfering with miR-NC, miR-34a, miR-34a plus LV-Vector or miR-34a plus LV-Nampt. **e**–**g** Determination of NAD^+^ content (**e**) and NAD^+^/NADH ratio (**f**), as well as Sirt1 deacetylase activity (**g**) after interfering with miR-34a expression; *n* = 3 independent experiments. **P < 0.01 vs. miR-NC, miR-34a, P10 anti-NC, or O anti-NC; ^*△*^*P* < 0.01 vs. miR-34a, ^*▲*^*P* < 0.01 vs. miR-34a plus LV-Vector

To gain deeper insights into the mechanism underlying how miR-34a instigated MSC senescence, we next detected senescent-associated phenotypes following miR-34a overexpression and co-transfection of miR-34a repletion with lentivirus expressing Nampt (LV-Nampt) or with its lentiviral vector (LV-Vector) in young P3MSCs. SA-β-gal staining (Fig. 7a) and quantitative analysis (Fig. 7b) revealed that, in comparison with the miR-NC group, SA-β-gal activity was strikingly more abundant in miR-34a over-expressed P3MSCs, but later, the SA-β-gal activity was noticeably downregulated in response to Nampt repletion. Additionally, the supplementation of Nampt could not only significantly antagonized the effect of miR-34a-induced diminished Nampt expression in P3MSCs (Fig. 7c), but could also downregulated the elevated levels of p16^INK4a^ expression caused by miR-34a repletion (Fig. 7d). These results illustrated that the strong induction of MSC senescence instigated by miR-34a sufficiency can be obviously recovered by Nampt repletion, further indicating that miR-34a affects MSC senescence through directly interacting with Nampt. It has been validated that Nampt can postpone both MSC replicative and natural senescence by mediating the NAD^+^-Sirt1 axis [10, 24]. To further prove that miR-34a-mediated MSC senescence by targeting Nampt is relevant to NAD^+^-Sirt1 signaling, we assessed the effects of miR-34a repletion and miR-34a depletion on intracellular NAD^+^ content, NAD^+^/NADH ratio, and Sirt1 deacetylase activity. From these results, we found that miR-34a overexpression in P3MSCs led to prominent reductions in the NAD^+^ content (Fig. 7e), as well as NAD^+^/NADH ratio (Fig. 7f), and Sirt1 activity (Fig. 7g). On the contrary, miR-34a suppression in either P10MSCs or OMSCS contributed to conspicuously higher NAD^+^ content (Fig. 7e), NAD^+^/NADH ratio (Fig. 7f) and Sirt1 activity (Fig.7g) than those in the control group (P10 anti-NC or O anti-NC). Collectively, the aforementioned results support the view that miR-34a-induced senescence can be rescued by Nampt replenishment via preserving NAD^+^ biosynthesis and Sirt1 activity, further verifying that miR-34a exacerbates MSC senescence by directly targeting Nampt and then mediating NAD^+^-Sirt1 signaling pathway.

## Discussion

The evolutionarily conserved miR-34 family comprises three homologous genes, miR-34a, miR-34b, and miR-34c. As evidence for modulatory role of miR-34 in senescence is growing [27, 28], we concentrated on this three-member miRNA family. On the one hand, we showed that the expression levels of miR-34 family members increased progressively during the process of consecutive passages in vitro. Of note, miR-34a expressed much more abundant than miR-34b/c, which is similar to the results of Ho Park et al [29]. On the other hand, we found that senescence in MSCs derived from aged rats contributed to the different degrees of increase in miR-34 family levels, and particularly, miR-34a expression elevated the most prominently as compared to that in young controls. These data were congruent with the findings of Boon et al [15]. Hence, we confirmed the upregulation of miR-34 family members in senescent MSCs, and among which miR-34a was the predominantly expressed one and presented a significant passage-dependent and age-dependent elevation.

MiR-34a, located in the region of chromosome 1p36 and encoded by its own transcript, is originally identified as a p53 responsive miRNA [30]. Recently, miR-34a has increasingly emerged as a potent posttranscriptional regulator in orchestrating aging process and cellular senescence [14, 15, 31]. In addition**,** ample evidences have confirmed elevated miR-34a levels were induced in various cells, tissues, and organs, including senescent endothelial progenitor cells (EPCs), lung myofibroblasts in both human and mouse [32], hearts of aged mice [15], and liver, kidney, and brain of old rats [33–35]. In accordance to these data, herein, we unveiled that miR-34a levels were higher to a varying degree in the hearts, brains, livers, kidneys, and lungs of old rats than those of the young ones, and especially enriched in the hearts. The discrepant findings in miR-34a expression between different cell types and tissues might be ascribed to the biological distinction of different species or tissue-specific expression of miRNAs in different developmental stages. On account of these, we focused on miR-34a instead of miR-34b/c in this report and speculate that miR-34a might play a pivotal regulatory role in MSC replicative and natural senescence.

To test our speculation of miR-34a crucially contributing to both the two types of MSC senescence, we modulated miR-34a expression via gene manipulation in young and senescent MSCs. As expected, we observed that miR-34a overexpression in young P3MSCs markedly induced senescence-related alterations, including senescence-like morphology, declined cell proliferation capacity, and retarded cell cycle progression with the majority of cells arrested in G1 phase. With regard to osteogenic differentiation potency is another salient property of SCs, we set out to determine whether the changes of miR-34a expression influence it. Ho Park et al. reported that miR-34a treatment of human adipose tissue-derived SCs (ADSCs) prominently reduced their osteogenesis, and this reduction was recovered by co-treatment with anti-miR-34a, indicating that miR-34a could be significant in determining the osteogenesis potency of ADSCs [29]. Consistent herewith, we displayed that miR-34a repletion impaired osteogenic differentiation capacity, whereas miR-34a depletion improved it. Besides, the results of Li Chen et al. also indicated that miR-34a was a negative regulator of osteoblast (OB) differentiation in human ADSCs, since its overexpression consistently resulted in attenuated in vitro OB differentiation and in vivo bone formation, and its silencing led to the converse effects [36]. Nevertheless, our finding is at variance with a recently reported scenario where miR-34a overexpression exerted promotion effects on the osteogenic differentiation potential of human adipose-derived stem cells (hASCs) both in vitro and in vivo, while miR-34a knockdown resulted in the opposite tendency [37]. The discrepant observations might owe to the different sources of SCs and the uncontrollability of the microenvironment, such as the divergent cell culture conditions and the discrepancy between model species.

To verify the occurrence of senescence in miR-34a over-expressed young MSCs, we firstly performed SA-β-gal staining at the cellular level. The lysosomal SA-β-gal activity accumulated more abundant in miR-34a sufficient group than miR-NC group, implying that miR-34a replenishment could induce or accelerate senescence of young MSCs. Cyclin-dependent kinase inhibitors p16^INK4A^ and p21^WAF1/CIP^ are reliable biomarkers to identify senescent cells [38–40]. Ju Li et al. reported that the expression of both p16^INK4A^ and p21^WAF1/CIP^ were augmented in aged muscle SCs in comparison with their levels in young cells [39]. Previously, we have found that both senescence-associated factors p16^INK4A^ and p21^WAF1/CIP^ levels were increased in natural senescent MSCs acquired from old rats as compared to their levels in MSCs from young controls, with p16^INK4A^ expression increasing much more dramatically [10]. Moreover, in spite of the significantly upregulated p16^INK4A^ mRNA expression levels exhibited in replicative senescent MSCs, inconspicuous alteration in p21^WAF1/CIP^ mRNA expression was observed following serial passages [24]. In good agreement with our previous studies, we herein manifested that, in miR-34a over-expressed young P3MSCs, p16^INK4a^ levels heightened markedly, whereas changes of p21^WAF1/CIP^ mRNA levels were unnoticeable; in natural senescent OMSCs, both p16^INK4a^ and p21^WAF1/CIP^ expression presented significantly downward trend after miR-34a silencing; and in replicative senescent P10MSCs, p16^INK4a^ levels dramatically downregulated; however, p21^WAF1/CIP^ levels were unchanged after the anti-miR-34a treatment. And the reason why p16^INK4A^ and p21^WAF1/CIP^ expressed inconsistently might be attributed to the discrepancy in the different regulatory mechanism concerning natural senescence and replicative senescence, concretely, the possibility of activating either or both the canonical p53/p21 and p16/pRb pathway, which are considered as the final effectors of the senescence program, and thus further explorations are warranted [41, 42]. Consequently, these data yielded the finding that miR-34a repletion can accelerate senescence-related variations of young P3MSCs. In addition, we analyzed miR-34a-deficient P10MSCs and OMSCs as well. In contrast to repletion, specific depletion of miR-34a in senescent MSCs can ameliorate replicative and natural senescence. Thus, we provide a proof of concept that miR-34a exerts regulatory functions on both MSC replicative senescence and natural senescence.

Along with the MSC replicative and natural senescence, we found intriguingly that Nampt expression decreased whereas miR-34a expression inversely incremented. And considered the miRNA-target prediction analysis predicting that there exist potential seed-matching sites between miR-34a and the 3′UTR of Nampt mRNA, whether Nampt is precisely regulated by miR-34a in MSC senescence still remains elusive. To this end, we then performed the luciferase reporter assay and the result verified that miR-34a specifically bound and interacted with its potential target Nampt, which was consistent with the published findings [14, 43]. In parallel, we determined the effect of miR-34a on alterations of Nampt expression levels in both young and senescent MSCs. Our findings confirmed that miR-34a could directly interact with Nampt and negatively regulates Nampt. Subsequently, we certified that miR-34a-induced senescence in young P3MSCs could be rescued by Nampt restoration, as evidenced from the significant alleviation of miR-34a-induced augmented β-gal-positive cells and the obvious downregulated expression levels of Nampt and p16^INK4A^. Here, our results further highlight the notion that miR-34a could functionally influence MSC senescence by directly targeting Nampt.

Energy metabolism dysfunction is one of the molecular bases of cellular senescence. The stem cell lifecycle—from acquisition and maintenance of stemness to lineage determination—is gradually recognized as a metabolism-dependent process [44]. NAD^+^ depletion has emerged as a elementary feature of aging that may predispose to a wide range of age-associated disorders, such as cancer, diabetes mellitus, and Alzheimer’s and Parkinson’s disease [45, 46]. Studies have shown that senescent cells have undergone significant metabolic changes [47, 48], among which NAD^+^, acting as a critical coenzyme in cellular energy conversion, participates in major energy production pathways, such as glycolysis, oxidative phosphorylation (OxPhos), and tricarboxylic acid (TCA) cycle [49]. Sirt1, serving as cellular sensor to monitor energy availability and regulate metabolic processes, is central to the control of metabolic processes and its function is intrinsically linked to cellular metabolism [50, 51]. And Sirt1 can be activated by high NAD^+^ levels, a condition caused by low cellular energy status, thus NAD^+^-SIRT1 pathway playing a pivotal role in increasing cellular energy stores and eventually maintaining cellular energy homeostasis, affecting cellular senescence [44, 52].

Moreover, in the Nampt-NAD^+^-Sirt1 axis, Nampt indirectly modulates Sirt1 deacetylase activity via affecting NAD^+^ biosynthesis, and subsequently, Sirt1 regulates a large number of age-associated signaling molecules downstream by deacetylation, thereby exerting a pivotal influence on individual aging and cellular senescence [20, 53]. Currently, NAD^+^ level as well as NAD^+^/NADH ratio was reduced, and Sirt1 activity was attenuated upon interference with miR-34a overexpression in young MSCs. On the contrary, miR-34a suppression contributed opposite effects to senescent MSCs. These results were in accordance with the published data displaying that the levels of miR-34a expression augmented in obese mice, thus leading to the reduced hepatic NAD^+^ content and Sirt1 activity via targeting Nampt, which also indicated that diminished Nampt expression contributed to miR-34a-induced hepatic NAD^+^ insufficiency in obesity [14].

## Conclusions

Our data validate that miR-34a exerts regulatory effects on MSC senescence, containing replicative senescence and natural senescence, by directly targeting downstream Nampt. Further, our study for the first time highlights that Nampt-mediated NAD^+^ biosynthesis and Sirt1 deacetylase activity are critical determinants in miR-34a-mediated MSC senescence. Our study deciphers the mechanism implicated in miR-34a-Nampt network modulating MSC senescence from the perspective of epigenetic regulation, which may not only potentially enrich insights into the molecular mechanism underlying MSC senescence, but may also afford a new stem cell rejuvenation approach and possibly a novel targeted therapeutic strategy for the prevention or treatment of age-associated disorders. Nevertheless, deeper studies on delineating miR-34a-mediated functions by in vivo approach as well as other epigenetic mechanism governing interactions of miR-34a-Nampt network on MSC senescence are warranted to be confirmed later, for there are other miRNA-target gene regulatory machinery or other senescence-associated long non-coding RNAs (lncRNAs) or circular RNAs (circRNAs) that might be also involved. Correspondingly, investigations are needed to unravel these issues to further benefit future applications of MSCs.

## Data Availability

All relevant data are within this paper.

## Abbreviations

MSCs: Mesenchymal stem cells
miR-34a: MicroRNA 34a
miRNA: MicroRNA
lncRNA: Long non-coding RNA
circRNA: Circular RNA
3′UTR: 3′Untranslated region
NAD: Nicotinamide adenine dinucleotide
Nampt: Nicotinamide phosphoribosyltransferase
Sirt1: Silent information regulator 2 ortholog
SA-β-gal: Senescence-associated-β-galactosidase
RT-qPCR: Real-time quantitative polymerase chain reaction
EGFP: Enhanced green fluorescent protein
NC: Negative control
anti-34a: Anti-sense miR-34a
